# Spatial Smoothing Effect on Group-Level Functional Connectivity during Resting and Task-Based fMRI

**DOI:** 10.3390/s23135866

**Published:** 2023-06-24

**Authors:** Cemre Candemir

**Affiliations:** International Computer Institute, Ege University, Izmir 35100, Turkey; cemre.candemir@ege.edu.tr

**Keywords:** connectivity analysis, fMRI, functional connectivity (FC), full-width half maximum (*FWHM*), gaussian kernel, resting state, task-based fMRI, spatial smoothing, smoothing effect

## Abstract

Spatial smoothing is a preprocessing step applied to neuroimaging data to enhance data quality by reducing noise and artifacts. However, selecting an appropriate smoothing kernel size can be challenging as it can lead to undesired alterations in final images and functional connectivity networks. However, there is no sufficient information about the effects of the Gaussian kernel size on group-level results for different cases yet. This study investigates the influence of kernel size on functional connectivity networks and network parameters in whole-brain rs-fMRI and tb-fMRI analyses of healthy adults. The analysis includes {0, 2, 4, 6, 8, 10} mm kernels, commonly used in practical analyses, covering all major brain networks. Graph theoretical measures such as betweenness centrality, global/local efficiency, clustering coefficient, and average path length are examined for each kernel. Additionally, principal component analysis (PCA) and independent component analysis (ICA) parameters, namely kurtosis and skewness, are evaluated for the functional images. The findings demonstrate that kernel size directly affects node connections, resulting in modifications to functional network structures and PCA/ICA parameters. However, network metrics exhibit greater resilience to these changes.

## 1. Introduction

Although the human brain weighs only about 1.35 kg (~3 pounds) on average and consists of fat, water, and protein, the working mechanism is the most complex one among the known structures [1]. The developments in neuroimaging techniques in recent decades have gained a fast acceleration on the working mechanism of the human brain. One of the leading techniques is functional magnetic resonance imaging (fMRI), which captures the change in the blood oxygen levels in the brain and creates a related time series of images [2]. In general, the studies on functional imaging can be categorized into two main perspectives: (i) resting-state fMRI (rs-fMRI) and (ii) task-based (also known as task-related or task-evoked) fMRI (tb-fMRI) [3]. The rs-fMRI focuses on the intrinsic neuronal activity, which reflects the spontaneous fluctuations while the subject is at “rest”. On the other hand, tb-fMRI focuses on extrinsic neuronal activity, unlike rs-fMRI. As opposed to intrinsic activity, extrinsic activity reflects the signals that arise from performing some given tasks during the scan. The task may show great variety according to the hypothesis, from motor movement to speaking or from making calculations to internally rehearsing a skill [4,5]. Until recent decades, the main aim was to map the specific brain areas with the given stimuli type and associate the task-responsible regions. This characterization, also known as localization, requires carefully designed special fMRI tasks, which are performed by the subjects in the MR scanner [6]. Nevertheless, the most current paradigms in neuroscience indicate that cognitive tasks are performed not only by individual brain regions working alone but also by networks of several separate brain regions that are functionally connected [7,8]. Thanks to the graph theoretical approaches, the brain can be modeled as a highly complex network, defined with brain regions as nodes and connections as links [9,10,11]. Functional connectivity offers a great way to explore the communication among the distinct brain regions and information passing on to each other [12,13,14,15]. Essentially, it is based on measuring the similarity of the blood-oxygen-level-dependent (BOLD) signals between selected seeds, i.e., regions-of-interest (ROIs) or voxels. If the signals are similar and correlated, it tends to mean that the seeds are functionally connected. Functional connectivity studies on rs-fMRI reveal a good way of understanding network connectivity in health and disease, while studies on tb-fMRI help explore the functional organization in the stimulated human brain.

Even though fMRI offers great benefits, it is also well known for having some drawbacks. The major problem is that the BOLD signals have very noisy structures, resulting in a low signal-to-noise ratio (SNR) [16]. Regardless of the fMRI type, i.e., either rs-fMRI or tb-fMRI, each voxel contains artifacts caused by psychological reasons such as breathing, cardiac pulsations, or scanner-related issues. This may lead a great inter-subject variability and becomes a great challenge when the task is more subject-specific, for example, memory or emotion, especially in multi-subject studies [17]. In order to overcome this problem and increase the SNR value, a smoothing step is applied to the raw functional data commonly, in addition to the other preprocessing steps (realignment, co-registration, slice-timing, and normalization). Here, it is worth noting that a common gold-standard preprocessing flowchart has not yet been reported [18,19]. Since the fMRI analysis is a highly data-driven method based on the BOLD responses of the voxels, the parameters of the preprocessing may result in great differences in the processed signals [20,21,22,23,24]. Thus, it is crucial to apply the same selected procedure for preprocessing of each subject. Regardless of which steps are followed in preprocessing, the key point is not to overlook how the selected parameters may affect the functional data.

In this study, we focused on smoothing, which is one of the most important components of preprocessing. It is implemented by the Gaussian kernel and defined by full-width-half-maximum (*FWHM*) [25]. Primarily, during the smoothing, each voxel value is replaced with the weighted average of the multiple neighboring voxels. While averaging yields reduced noise, i.e., increased SNR value, it also decreases the spatial resolution, i.e., it blurs the image, may remove the active voxels, and causes merged or split activation clusters [26,27]. This may be problematic when the cluster size of the ROIs is too small, such as the amygdala, so that the voxels can contain some parts of the surrounding regions and vice versa [28]. Additionally, this also yields a great challenge in functional connectivity analysis. The related studies show that the size of the kernel, i.e., the smoothing level, affects the connectivity in terms of increasing the connection power or, on the contrary, decreasing the differences between connected voxels [29]. This results in either an increased correlation or increased artifacts on the voxels [30]. It is also reported that the selection of inappropriate kernel size decreases test–retest reliability [31]. Thus, it should pay attention to the trade-offs since it has a powerful impact on the signals. Nevertheless, it has been shown that smoothing also benefits the efficiency of the analysis [32]. Here, the critical point is to pay attention to the data-specific selection of the kernel size instead of a standard size. Additionally, the size is directly related to the region proposed in the research hypothesis. Although there are studies that analyzed this issue either in the aspect of independent component analysis and functional connectivity [33], inter-subject correlation [34], and functional brain networks [35], they are limited to analyzing only one condition at the same time: either being conducted with single-subject or analyzing rs-fMRI or else tb-fMRI. In summary, there are no studies to this extent on results demonstrating group-level effects of kernel size for both rs-fMRI and tb-fMRI simultaneously. In this respect, the information to be obtained from a comprehensive evaluation, including all the situations mentioned, has a high practical value in practice.

**Figure 1:**
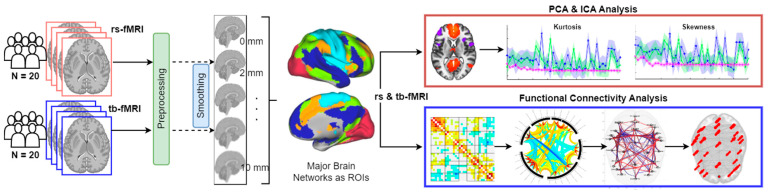
The graphical illustration of the effect of various kernel sizes at smoothing on fMRI data. To observe the effects, two fMRI datasets were used: resting state (rs-fMRI) and task-based (tb-fMRI) with n = 20 subjects each. For each subject, the same preprocessing steps were followed, and smoothing was performed with fwhm = {0, 2, 4, 6, 8, 10} mm. For whole brain coverage, the major networks were chosen as ROIs for each image. Afterward, group-level performances of rs-fMRI and tb-fMRI are analyzed in two-fold, separately. First, PCA and ICA decompositions were performed for each kernel size, and the changes in two main components, kurtosis and skewness, were measured. Second, functional connectivity analyses were performed to be able to see the network changes. Finally, the graph theoretical measurements were calculated for each size of kernels.

In this study, we explore how the smoothing kernel sizes affect the functional connectivity networks and network parameters of the whole brain with a multi-subject and multi-state (rs/tb-fMRI) study. For this purpose, we present a comprehensive analysis of the group-level effects of smoothing on functional connectivity networks during rs-fMRI and tb-fMRI. Therefore, we used two functional datasets that were acquired from a single MRI scanner (see Section 2 for details) of 20 healthy volunteers. For each of the subjects in the dataset, we constructed the functional connectivity maps using the {0, 2, 4, 6, 8, 10} mm Gaussian kernels and measured the smoothing effects as illustrated in Figure 1. The contributions can be summarized as follows:

1. We constructed the functional connectivity maps for both rs-fMRI and tb-fMRI in detail for each subject. For the whole brain coverage, all major brain networks (Default Mode Network (DMN), Somatomotor Network (SMN), Visual Network (VN), Salience Network (SN), Dorsal Attention Network (DAN), Frontoparietal Network (FPN), Limbic Network (LN), Cerebellar Network (CN) were included to the analysis. To the best of our knowledge, there is no such study that analyzes the functional interactions of all these networks together.
2. Although there are several studies that investigate the smoothing effect on the functional connectivity [30] or tb-fMRI [34] in single-subject [33] or on the healthy and diseased groups [35], however, to the best of our knowledge, there are no studies that have performed rs-fMRI and tb-fMRI together in such detail. Moreover, another important issue that differs from the other studies is that the functional images in the dataset involve sequential resting and task images that belong to the same subjects acquired from a single scanner. Thus, the dataset does not include intra-scanner and subject-related artifacts, which allows for observing the changes clearly.
3. The main graph metrics, betweenness centrality, global and local efficiency, clustering coefficient, and average path length, are also analyzed for each smoothing level both for rs-fMRI and tb-fMRI.
4. Besides the functional connectivity analysis, the main component of independent component analysis (ICA) and principal component analysis (PCA), in terms of kurtosis and skewness, are also investigated in detail for both rs-fMRI and tb-fMRI at the group level. We could not find similar studies during the literature search.

The rest of the paper is organized as follows: Dataset, imaging procedure, and data preprocessing are presented in Section 2. The methods, voxel-based analysis, and ROI-based analysis are presented in Section 3. The comparative results and conclusion are given in Section 4 and Section 5, respectively.

## 2. Materials

### 2.1. Data Preliminaries

**Figure 2:**
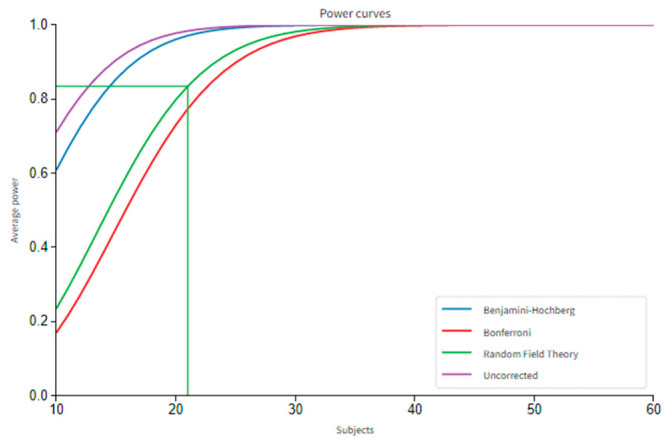
The power analysis graph for the subject group of the study.

rs-fMRI offers great benefits, such as requiring only the MRI scanner, not requiring the task-related programs, and other information beyond the resting, i.e., behavioral task-related responses. Through the advantages, it could be applicable to large subject populations and even to subjects who could not perform any tasks for any reason. On the other hand, tb-fMRI requires carefully designed tasks, so it is more laborious, and the number of such studies is relatively fewer than the rs-fMRI. In this study, to be able to seek the effect of *FWHM* kernel size differences on functional connectivity networks, a collection of datasets that consists of a sequential scan with rs-fMRI and memory fMRI task (mem-fMRI) was used. The structural and functional images were acquired by a 3T Siemens Magnetom whole-body MRI scanner at Ege University, Izmir, Turkey. The task was conducted by the Socat Lab research team. The whole research and task were approved by the subjects and the ethical committee of the university. In total, 30 volunteers without reported any diseases were involved in the scans. It was observed that they all had a normal or corrected vision at the initial examination. The exclusion criteria for selecting the subjects were having a mental illness, mental trauma in the present or in the past, having a medical disease such as tension, diabetes, etc., and having an Alzheimer’s story in the family). After the quality checks of the acquired data, 23 subjects (20–25 years old, mean = 23.35 ± 1.04, *f:*12 *m:*11) were involved in further analyses, while the others were discarded due to the high motion rates and incompleteness of the task. The power of the population was measured over 80% with the power analysis, which is given in Figure 2. According to the results of the power analysis, it was observed that nearly 21 subjects are enough to achieve power over 80%, according to the Random Field Theory (the commonly used scale). Since different tasks will create activations in different areas and levels in the brain, power analysis should be performed task-specifically in every study before further analysis. One recent study analyzed the required sample size estimation on three different functional datasets with power analysis in detail [36].

The parameters of the whole brain T2*-weighted EPI sequence are as follows: Repetition time (TR) = 3 s, echo time (TE) = 30 ms, voxel size 3×3×3 mm, matrix size = 64×64 with 37 slices, FOV=192×192 mm, flip angle (FA) = 90°. The parameters of the structural data were also acquired with 3D GR\IR T1-weighted images with matrix size 512×512×160, TR = 1600 ms, TE = 2.21 ms, slice thickness = 1 mm, voxel size 1×1×1 mm.

### 2.2. fMRI Task

The mem-fMRI is a block design fMRI task that consists of sequential rest and task phases. The fMRI procedure begins with the resting-state scan, in which the subjects are asked to rest with closed eyes for 300 s. Then, the task block starts, and during the task phase, a set of neutral faces and their names are shown on the screen for 6 s and a one-second black screen comes after. During the screens of 6 s, subjects are asked to memorize the name and face pairs. In total, 24 different face and name pairs are shown to the subjects in the encoding block in 288 s. Each period starts with a baseline screen (30 s) in task blocks. The face dataset used in the memory task is a subset of the FACES dataset, which only includes neutral face expressions [37]. 

It can be said that the main difference between this dataset and similar ones is conducted with a single MRI scanner in a single session for whole participants. Thus, both the scanner-related artifacts, such as calibration, noise, slice gap, etc., and user-related, such as MR technician, differences are eliminated. Since the scan is acquired in one session, the conditions of each participant are guaranteed, such as being in the same physiological conditions or attention. These aspects allow for the investigation of the functional data without any external effects.

### 2.3. Data Preprocessing

The preprocessing of rs-fMRI and tb-fMRI data was performed by using CONN (version 21.a) functional connectivity toolbox [38]. During the preprocessing, common steps were followed as a default preprocessing pipeline for each subject. All functional images were realigned and corrected for head motion (as stated in Section II-A, 3 subjects were discarded at this step). Afterward, all slices were aligned at the slice timing step so that they were synchronized temporally. The pipeline then continued with the co-registration step, in which the anatomical scans of the subjects were registered to the mean of the images. The segmentation step came after, in which the brain was separated into surrounding tissues, and the white matter and CSF were extracted. During the segmentation, the anatomical images were also registered to the MNI-152 (Montreal Neurological Institute) standard space T1-weighted average structural template for further group analysis. Finally, the spatial smoothing step was applied for all rs-fMRI and tb-fMRI images with isotropic Gaussian kernel at the different sizes: *FWHM* = {0 (as reference for no smoothing), 2, 4, 6, 8, 10} mm, which are the most frequently used in practice. The smoothing was consistently applied before calculating the functional connectivity networks, ICA, and PCA components extraction with each and every parameter set. Additionally, for each *FWHM* value, quality checks of the processed data were performed.

## 3. Methods

### 3.1. Smoothing

The primary aim of smoothing is to reduce the noise, which increases the SNR value in the BOLD signal and minimizes the errors in group-level analysis. Commonly, it is applied as the last step of the preprocessing pipeline. As we stated above, it has several good benefits as well as detrimental effects. A typical spatial smoothing is evaluated by a Gaussian kernel, and the smoothing step results in each voxel being transformed into the weighted average of its nearby voxels. The averaging process is performed by applying a smoothing filter, which represents the smoothing matrix. The width of the kernel is determined by full-width-half-maximum (*FWHM*) [25]. The relationship between the *FWHM* and the standard deviation of the Gaussian kernel can be shown as follows:

(1) $$\sigma =\frac{FWHM}{2\sqrt{2\ln\left(2\right)}}\;\;\;$$

The width of the kernel is w, and it is symmetric between [−0.5 w and 0.5 w]. Due to the relation between the *FWHM* and standard deviation, (1) can be rewritten as follows:

(2) $$w=\frac{6\sqrt{2\ln\left(2\right)}}{\pi }\cdot \frac{1}{FWHM}$$

Regarding (2), for a voxel *i*, the smoothing operation with its nearby voxels can be defined as follows:

(3) $$x_{i}=\frac{\sum_{k}w_{i}\left(k\right)\cdot x_{k}}{\sum_{k}w_{i}\left(k\right)}\;\;\;\;\;$$

Here, $x_{i}$ represents the time series of the $i^{th}$ voxel, and $w_{i}\left(k\right)$ denotes the value of the $k^{th}$ voxel on which the kernel is centered at the $i^{th}$ voxel. It is obvious that as the kernel size becomes wider, the smoothing filter contains more neighboring voxels inside and vice versa. There are several common assumptions and recommendations that the *FWHM* kernel size should be 1.5–2 times, or three times the voxel size (i.e., applying *FWHM* = 3–6 mm smoothing for a voxel size of 2 × 2 × 2 mm) [7,39]. The other concept is not to choose a kernel size wider than the ROI. For example, if the ROI is as small as the amygdala, the *FWHM* size should be set smaller than the size of it, while if the ROI is a subcortical motor region, the *FWHM* size may be set to 8–10 mm. Despite the second recommendation being more common, there is no unique consensus on it.

We applied five kernels in total at sizes {2, 4, 6, 8, 10} mm and 0 mm kernels (as the reference for non-smoothing). These kernel sizes are in the commonly recommended range in the literature; however, it is not yet obviously clear how these Gaussian sizes affect the functional connectivity networks and their related parameters.

### 3.2. Functional Connectivity Network Extraction

Thanks to the graph theory, the brain can be modeled as a network regarding its connectivity properties. The term connectivity can refer the regional connectivity, also known as anatomical or structural connectivity, which defines the short-distance connections between nearby voxels, or effective connectivity, which defines the causal effects [13]. On the other hand, it is also possible to define functional connectivity, which refers to the indirect connections between discrete or spatially distant brain regions. Functional connectivity measures the correlation or, in other terms, dependencies of the BOLD signals among the different areas. The structural and effective connectivity is out of scope in this manuscript. 

**Figure 3:**
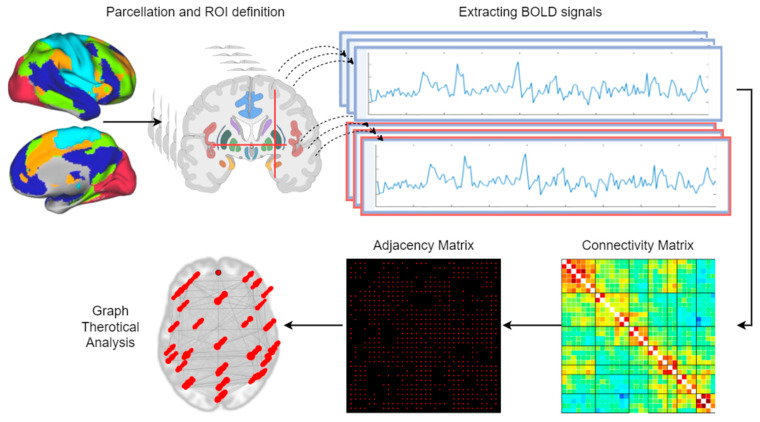
Flow of the graph theoretical analysis of functional connectivity. After the ROI selection, the time courses of the BOLD signals are extracted, and correlation analyses are performed. Connectivity matrices are constructed according to the correlation coefficients. Later, the values are often thresholded and then binarized to obtain the adjacency matrices that define the connections between nodes. The connections can be visualized as a graph where ROIs denote the nodes and the associations denote the edges. Then, topological network metrics such as global efficiency or average path length can be measured on the final graph.

A functional network is interpreted with nodes and edges in graph modeling, where nodes are the disjoint brain regions, and edges are the connections or connection strengths between the nodes. Formally, the network can be described as a graph *G*(*N*,*E*)*,* with N representing the set of nodes and *E* being the edges in the graph. Once graph *G* is defined, the connectivity pattern can be designated by a symmetric adjacency matrix sized N×N, and the connectivity matrix can be acquired. As illustrated in Figure 3, the common connectivity analysis consists of the following steps: After the preprocessing, i.e., the initial step for all functional data, the node definition (or parcellation) or seed selection should be made. For this step, either the atlases, such as automated anatomical labeling (AAL), Harvard–Oxford atlas, or manually defined subject-specific or task-specific ROIs can be used. In this way, voxels are grouped into areas that are considered as functionally homogeneous. Afterward, the BOLD signals, which are the time series of the voxels, are extracted for each node within each region. The third step is calculating the correlations for extracted time series. The correlation coefficients (*r*) are computed for each subject separately and converted into Fisher’s z-transformation. *r*-to-*z* transformation can be defined as follows [40]:

(4) $$r=\frac{cov\left(X,Y\right)}{\sigma_{X}\sigma_{Y}\;},\;\;\;\;z_{r}=\frac{1}{2}\ln\left(\frac{1+r}{1-r}\right)=\mathrm{arctanh}\left(r\right)\;\;\;\;\;$$

Since the correlation is not normally distributed, Fisher transformation allows for calculating the (r) in a confidence interval. Therefore, it is strongly recommended to make the values in the connectivity matrix approximately normally distributed [40]. Finally, the last step is building the N×N symmetric connectivity matrix with the values $r_{XY}$, where r, (namely $\left(z_{r}\right))$ denotes the correlation value between the voxel X and Y. Once the matrix has been acquired, a threshold value can be applied for the graph representation.

In this study, the nodes are defined as the set of major functional brain networks with their high-level cognitive areas [41] for whole brain coverage. These networks dominate cortical activity in the brain to the greatest extent, and they all perform related but distinct tasks. These major networks regulate brain activity both while the brain is at rest and during processing tasks. The neural networks of the brain operate in a hierarchical manner, integrating and synchronizing to carry out complicated tasks.

The major networks can be listed as Default Mode Network (DMN), Somatomotor Network (SMN), Visual Network (VN), Salience Network (SN), Dorsal Attention Network (DAN), Frontoparietal Network (FPN), Limbic Network (LN), and Cerebellar Network (CN). In a hierarchical manner, the sub-regions of the major networks can be listed as follows: \textit {N = {DMN.MPFC, DMN.LP(l-r), DMN.PCC, SMN.Lateral(l-r), SMN.Superior, VN.Medial, VN.Occipital, VN.Lateral(l-r), SN.ACC, SN.AInsula (l-r), SN.RPFC(l-r), SN.SMG(l-r), DAN.FEF(l-r), DAN.IPS(l-r), FPN.LPFC(l-r), FPN.PPC, LN.IFG(l-r), LN.pSTG(l-r), C.Anterior, C.Posterior}}. These sub-regions constitute the total node set (N = 32) in the analyses (see Appendix A for further detail).

**Figure 4:**
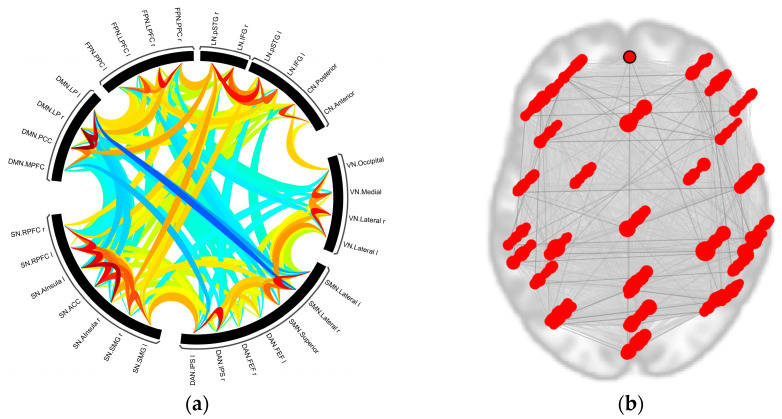
The functional connectivity map of the nodes: (**a**) The darker red lines show strong connectivity, whereas the blue lines show weaker connectivity in the connectome ring. (**b**) The topological locations of the determined nodes in the brain. The darker red lines show that even though the nodes settle in distinct regions, they are connected with strong functional connectivity.

A sample connectivity map of all nodes is given in Figure 4, where the red lines indicate the strong functional connections, and the blue lines denote the weaker connections. Here, it can be clearly seen that even though the nodes are located in distributed regions, they are still connected functionally.

### 3.3. Voxel-Based Analysis

The voxel-wised analyses are helpful when we explore the connectivity effect on the whole brain without the restriction of any a priori regions or selected ROIs. However, since correlation values for each and every combination of the voxels are calculated, the cost of this analysis is high. However, it can be reduced with the application of common, well-known decomposition methodologies so that how the underlying components are related to each other and how these components are estimated from the four-dimensional data. In this context, two best-known methodologies, principal component analysis (PCA) and independent component analysis (ICA), are investigated under the different smoothing kernels separately. They are both data-driven techniques used in fMRI data analysis for identifying patterns of brain activity. Here, it should be noted that PCA or ICA is not better than the other; they differ in their assumptions, objectives, and results. Each method has its own benefits, which are detailed below, and the choice of the decomposition methodology can change the research hypothesis.

#### 3.3.1. Principal Component Analysis (PCA)

One of the major challenges of fMRI data lies in being big data. Among the over 100.000 voxels, some of them may contain correlated or uncorrelated information. On the other hand, this becomes more problematic in terms of computational cost, memory restrictions, etc., when the datasets consist of large subject groups [30]. A common and powerful approach is applying principal component analysis (PCA) to compress the information, i.e., reduce the dimensionality. PCA is a linear method that seeks to identify the directions of maximal variance in a dataset. When we apply the PCA to fMRI datasets, the extracted components may contain Eigen images or spatial patterns whose variations are defined by their eigenvectors over time. The eigenvalue defines the variance explained by each component, and as the eigenvalue increases, it means that a larger variance could be explained. Since PCA is applied iteratively, it generates orthonormal components. The principal components are linear combinations of the original fMRI time series, and they can be interpreted as spatial maps of brain activity that represent different sources of neural activity in the brain. However, PCA is a completely data-driven method, so its success can also be directly affected by the smoothing level of the data. To explore the smoothing effect on PCA, first, we performed the group-level dimensionality reduction before the decomposition and then performed the other steps, such as back projection, for characterizing the voxel-to-voxel connectivity matrix. Nevertheless, this can be grueling and difficult to be able to decide which components should constitute the main component set, which can represent the original data when the others are discarded. Moreover, the extracted first components could be reflecting the noise due to the orthogonality, and these components also affect the following extracted components. At this point, ICA may provide a better approach in such circumstances due to its components should be independent as possible [42]. Here, “independent” refers to minimum overlaps between the components; thus, it can be applied effectively.

#### 3.3.2. Independent Component Analysis (ICA)

ICA, on the other hand, is a statistical method that seeks to identify statistically independent sources of brain activity. ICA assumes that the acquired BOLD signal consists of a mixture of various components that cannot be visually recognized but can be distinguished. These components are statistically independent and represent a different spatial pattern of brain activity that is not necessarily related to the spatial maps identified by PCA. Unlike the orthogonal vectors founded by PCA, ICA uses high-order statistics to maximize independence. A common ICA model is formulized by X=A.s, where X is the mixture (i.e., the data) and denoted by $X=\left(x_{1},\;x_{2},\ldots ,x_{m}\right)$, s is the N-dimensional independent source vector which is shown by $s=\left[s_{1},s_{2},\ldots ,s_{N}\right]$, and $A_{MxN}$ is the matrix of the mixing coefficients. Here, ICA aims to achieve the un-mixing matrix ${\hat{W}}_{MxN}$, so that the sources s could be estimated with y=W.X.

ICA is a data-driven and blind decomposition method that identifies the components based on the principle of non-Gaussianity. Thus, it is possible to identify the set of time courses and independent spatial maps that explain the data by finding the components that are maximally non-Gaussians [7]. In this way, it can also identify highly functionally connected networks. Similarly, it can be used to extract the functional connectivity matrix, such as cognitive, motor, or sensory networks, for a given stimulus. Another problem that ICA is used is to distinguish task-related and non-task-related components from the BOLD signals. The non-task-related components caused by the physiological behaviors, such as head motion or cardiac movements, can contribute to the activation signals as noise and creates artifacts. They may result in characteristic changes in the BOLD signal, so they should be removed or suppressed during the smoothing step in preprocessing. Here, it should be noted that the selection of Gaussian kernel size for smoothing constitutes a great variety for further ICA analysis because, in the ICA model, the artifacts are not modeled implicitly but are shown as separate components. 

In summary, PCA is used to reduce the dimensionality of the fMRI data and identify the sources of neural activity that explain the majority of the variance in the data, while ICA is used to identify independent sources of neural activity that may not be captured by PCA. Both methods can provide useful insights into the underlying patterns of brain activity in fMRI data and can be used in combination with other data analysis techniques.

### 3.4. ROI-Based Analysis

**Figure 5:**
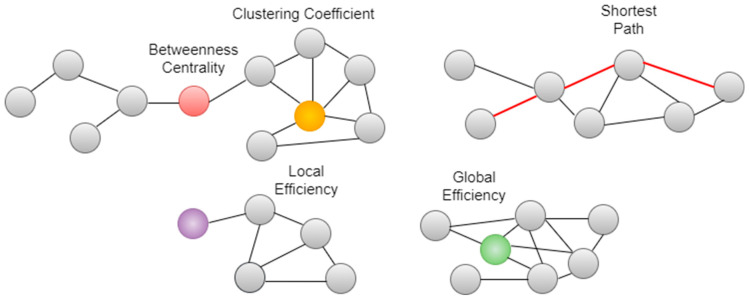
The representation of the graph measures.

Once ROIs, i.e., nodes, are defined, functional connectivity analysis of the whole set of nodes can be performed for each possible connection between all pairs of ROIs. The analyses give a statistical map that is estimated using a GLM for pairwise associations, characterizing the effect of interest in connectivity matrices. The F-statistics, which are acquired after the analysis, can tell whether there is any effect between all possible ROI pairs sensitively. Here, both with-in-network and between-network metrics, such as global efficiency, local efficiency, betweenness centrality, average path length, etc., can be calculated. Here, we focused on five main network metrics, which are global efficiency, local efficiency, betweenness centrality, average path length, and clustering coefficient. As illustrated in Figure 5, these metrics can be summarized as follows: Efficiency is a measure of information propagation and can be calculated both as global and local [43]. The global efficiency is the distant information transmission of all networks and is defined as the average of the inverse shortest path length between all node pairs:

(5) $$E_{glob}\left(G\right)=\frac{1}{N\left(N-1\right)}\underset{i\neq j\;\in G}{\sum }\frac{1}{d_{ij}}\;\;\;\;\;\;\;$$

where *G, K*, and *E* denote the network, nodes, and edges, respectively. $d_{ij}$ indicates the shortest path length between the nodes *i* and *j.* Here, please note that the path length between the nodes, i.e., the length of an edge, is interpreted as the correlation coefficient where a high correlation indicates a short length and vice versa. The local efficiency, on the other hand, is defined as the capability of information change in each subgraph and calculated as:

(6) $$E_{loc}\left(G\right)=\frac{1}{N}\underset{i\in G}{\sum }E_{glob}\left(G_{i}\right)\;\;\;\;\;\;\;\;$$

Here, the subgraph contains all neighboring nodes, which are directly connected to node *i.* $E_{loc}$ is thought of as the measure of the functional decomposition in such complex networks [44]. Betweenness centrality metrics would indicate a high score if it is located in many shortest paths; therefore, it is a measure of which nodes act as a bridge in the graph. Betweenness centrality is expressed as follows, where $\sigma_{ik}$ denotes the total shortest path number from node *i* to node *k* and $\sigma_{ik}\left(j\right)$ is the number of these paths passed through node j:

(7) $$BC\left(j\right)=\underset{i\neq j\neq k}{\sum }\frac{\sigma_{ik}\left(j\right)}{\sigma_{ik}}\;\;\;$$

The clustering coefficient CC(G) is a measure of which nodes are in connection to form a cluster together, i.e., it denotes the number of triangles in a graph. High CC(G) values indicate a dense network structure. Average path length l(G) is defined as the average number of steps along the shortest paths for all possible pairs of network nodes, which are formulated as:

(8) $$l\left(G\right)=\frac{1}{N\cdot \left(N-1\right)}\underset{i\neq j}{\sum }d_{ij}$$

The low l(g) and high CC(G) values are the two parameters of a small-world structured graph [45].

## 4. Results

### 4.1. Results for Voxel-Based Analysis: Effects on PCA and ICA Components

We analyzed two main parameters of PCA and ICA: kurtosis and skewness. Kurtosis indicates the degree of the outliers in the given signal or distribution. High kurtosis refers to having extreme tails, which means outliers, and low kurtosis refers to the light tails or absence of outliers. Skewness, on the other hand, is an indicator of symmetric distribution. A high skewness value indicates an asymmetrical distribution. If the data are skewed from either left (negative skew) or right (positive skew), a transform should be applied further until a normal distribution is reached because the tail could behave as an outlier in any case.

In the context of fMRI data analysis, kurtosis and skewness can be used to evaluate the distribution of the principal components obtained from PCA and independent components obtained from ICA.

In this study, PCA and ICA decompositions were performed with the CONN functional connectivity toolbox, which works on Matlab R2019b [38]. Since the selection/determination of the factor number for PCA and ICA decomposition is beyond the scope of the article, the recommended number of suitable components (n = 40) was automatically extracted.

**Figure 6:**
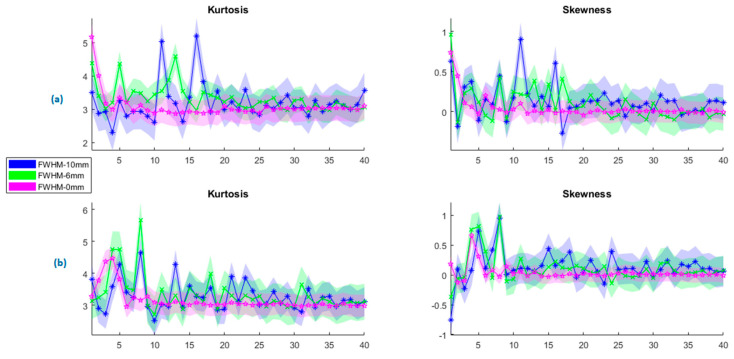
Change in the PCA components at different kernel sizes for (**a**) resting state (rs−fMRI) and (**b**) encoding state (tb−fMRI).

In Figure 6, the change in the kurtosis and skewness values of PCA are presented for both rs-fMRI and tb-fMRI at various FWHMs at sizes {0, 2, 4, 6, 8, 10} mm. In order to improve the clarity of the graphics, only minimum, average, and maximum values are given with their standard deviations. The pink, green, and blue regions show the fwhm = 0 mm (no smoothing), 6 mm, and 10 mm, respectively. Figure 6a shows the kurtosis and skewness parameters of PCA for the resting state, and Figure 6b shows the encoding state. Although the mean of kurtosis at rs-fMRI and tb-fMRI are nearly the same for fwhm values, the wider kernel size causes more outliers in rs-fMRI. A similar result can also be observed for the skewness parameter. The asymmetry of PCA components increases as the kernel size increases. Since the PCA does not constrain the distribution of the principal components, therefore they can be either symmetric or skewed according to the data characteristic.

On the other hand, ICA assumes that the sources of neural activity are statistically independent and non-Gaussian. Therefore, the independent components obtained from ICA are often more likely to have non-Gaussian distributions than the principal components obtained from PCA. In general, independent components tend to have higher kurtosis and skewness, reflecting their non-Gaussian nature.

**Figure 7:**
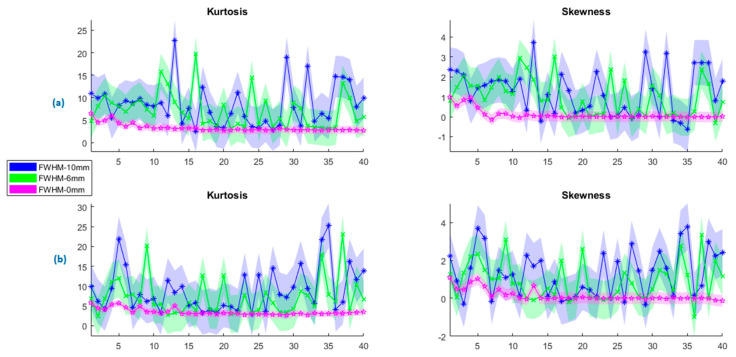
Change in the ICA components at different kernel sizes for (**a**) resting state (rs-fMRI) and (**b**) encoding state (tb-fMRI).

**Figure 8:**
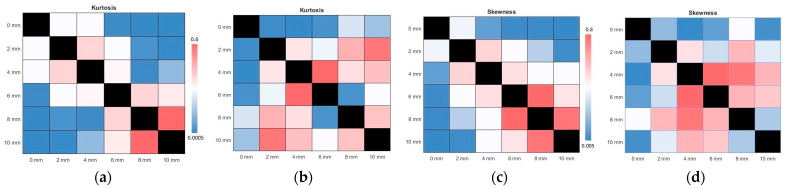

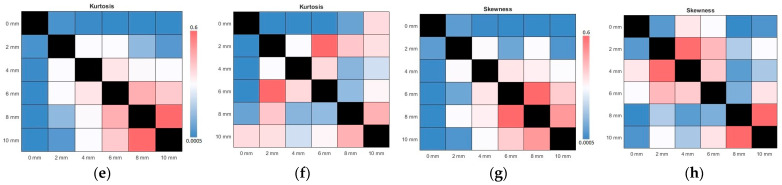
The results of analyses of ICA and PCA p-values for all fwhm kernel sizes in terms of kurtosis and skewness. The above line indicates the tb-fMRI results, whereas the below line shows the rs-fMRI results. (**a**,**b**) ICA and PCA kurtosis for tb-fMRI, respectively (**c**,**d**) ICA and PCA skewness of tb-fMRI (**e**,**f**) ICA and PCA kurtosis for rs-fMRI (**g**,**h**) ICA and PCA skewness of rs-fMRI. The color bars are common for consecutive images.

In parallel, the ICA results on the change in the kurtosis and skewness values for rs-fMRI and tb-fMRI are given in Figure 7. As the kernel size increases, the observed outlier behavior increases in ICA components. The mean of the kurtosis also tends to increase regularly for rs-FMRI and tb-fMRI. A similar observation can be performed for the skewness. If we skewed data with a wide kernel, it would diverge to the normal distribution, which also means an increased asymmetry. Here it should be noted that even though the non-smoothing gives the minimum results for kurtosis and skewness, it is not recommended to use PCA or ICA decomposition on non-smoothed data since the functional images are too noisy. As another analysis, the results of the *p*-value changes across all kernel sizes are given in Figure 8. It can be said that this recommendation is affirmed according to the given subfigures. The most significant *p*-value changes stand out in the 0 mm kernel size (i.e., non-smoothed data) when compared with the other kernel sizes on the top row (or first column). It confirms that the consecutive smaller kernel size changes may not reflect significant changes in these parameters, for example, changing the kernel size from 4 mm to 6 mm. However, the main difference can be observed better when it is changed, for example, from 0 mm to 6 mm.

It is worth noting that the interpretation of kurtosis and skewness in fMRI data analysis should be performed with caution, as these measures can be influenced by various factors, including the number of components, the specific data preprocessing steps, and the nature of the experimental design. Additionally, the choice of kernel size may also depend on the specific research question being addressed. For example, if the goal is to identify small, localized areas of activation, a smaller kernel size may be preferable. Conversely, if the goal is to identify larger, more diffuse areas of activation, a larger kernel size may be more appropriate.

The choice of kernel size can also depend on the anatomical features of the brain being studied. For example, a smaller kernel size may be used to smooth data from regions with a high degree of anatomical detail, such as the cortex, while a larger kernel size may be used to smooth data from regions with less detail. Therefore, it is important to carefully evaluate the distributional properties of the components obtained from PCA and ICA and to use additional methods to validate the results obtained from these techniques.

### 4.2. Results for ROI-Based Analysis: Effects on Functional Connectivity Networks

Graph theory analyses on functional connectivity networks are also performed with the CONN toolbox. For whole brain network analysis, ROIs (i.e., nodes) are defined as major brain networks and their high-level cognitive areas [41]. In total, 32 ROIs are determined for each participant both for rs-fMRI and tb-fMRI analysis (See Appendix A for the complete list of the ROIs). All correlation values were calculated, which constitute the weights (i.e., edges) of the functional connectivity matrices. Then, the Fisher transform was applied to make the values in the connectivity matrix approximately normally distributed. Four hundred and ninety-six significant connections were determined using an FDR-corrected threshold (*p* < 0.05). This procedure results in weighted matrices of size 32×32. Then, according to the presence of a connection, the matrices are converted into the binary adjacency matrix. 

**Figure 9:**
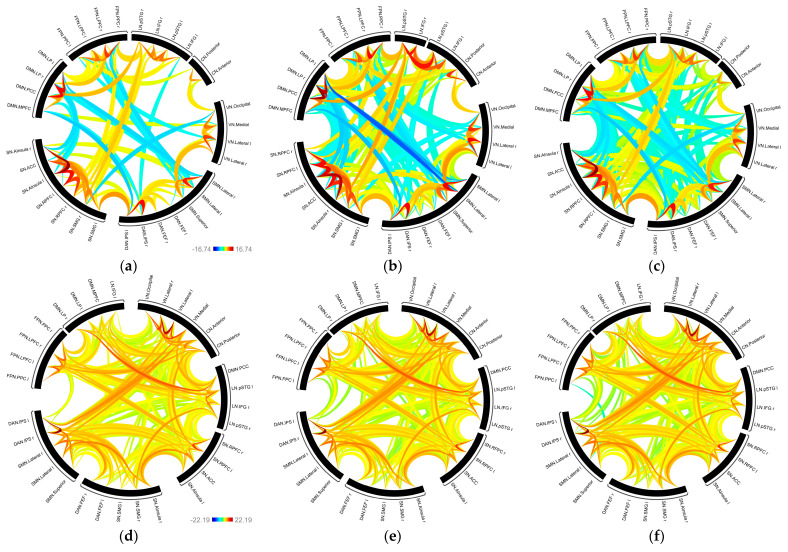
Functional connectivity networks as connectome ring for fwhm kernel sizes from left to right show 0, 6, and 10 mm, respectively: (**a**–**c**) resting state connectivities; (**d**–**f**) task-based connectivities. The color bars are common for all three images in each row.

First, the smoothing effect with different *FWHM* on rs-fMRI and tb-fMRI network structure is exhibited in Figure 9 (please see the Supplementary Materials for the connectome rings of all kernel sizes). Here, the circle denotes the selected nodes, and the links denote the network-based statistical results illustrated with their t-values. While red links show the strong positive functional relationships between the nodes, the blue links show the negative ones. The number and strength of the links differ as the kernel size changes, whereas the significant links tend to decrease as the kernel size increases. On the other hand, it can be clearly seen that rs-fMRI and tb-fMRI show great varieties among the connections in the graphics of minimum, average, and maximum kernel sizes, respectively. Later on, to explore the various smoothing parameter effects on topological properties, the above-mentioned metrics (global efficiency ($E_{glob}$), local efficiency ($E_{loc}$), betweenness centrality (BC), average path length ($l_{avg}$), and clustering coefficient (CC)) are computed on the binary adjacency matrix for both rs-fMRI and tb-fMRI. Group-level results are presented in Table 1.

Contrary to the functional connectivity results, it can be said that there are no significant statistical differences in the graph metrics at the different sizes of the smoothing kernels. On the other hand, the value of these metrics differs remarkably in rs-fMRI and tb-fMRI. While the values of $E_{glob},\;\;E_{loc},\;\mathrm{and}\;CC$ increased during the task state, the values of $BC\;\mathrm{and}\;\;l_{avg}$ metrics are decreased when compared to the resting state. However, in any case, the mean of the results shows that the best FWHMs are either 6 mm or 8 mm, which are commonly used Gaussian kernel sizes.

## 5. Conclusions and Future Work

The network properties of functionally connected brain networks may affect the various processing steps. Although gold-standard preprocessing has not been identified yet, definite consecutive steps are common. However, one of the fundamental steps, smoothing, is a data-driven process and should be decided specifically for the functional data.

In this study, we explored the topological functional network and parametric affections on different smoothing values at the preprocessing step of functional neuronal imaging data. In order to investigate the changes, functional images were smoothed at kernel sizes *FWHM* = {0, 2, 4, 6, 8, 10} mm. Both resting state and task state conditions are analyzed in the aspect of functional connectivity networks. For whole brain coverage, the major brain networks and their high cognitive areas were included in the analysis. First, we analyzed PCA and ICA decompositions with their main components: kurtosis and skewness changes. Then, functional connectivity networks of rs-fMRI and tb-fMRI were analyzed as functional connectome changes and the graph parameters: global efficiency, local efficiency, betweenness centrality, clustering coefficient, and average path length. Comparative results for PCA and ICA show that as the kernel size becomes wider, the observed outlier behavior increases in terms of kurtosis. The asymmetry, i.e., skewness, increases with the wider kernel size for all functional data. Nevertheless, rs-fMRI and tb-fMRI values show differences in functional connectivity relationships. The number and strength of the links differ as the kernel size changes, where the significant links tend to decrease as the kernel size increases both for rs-fMRI and tb-fMRI. However, it can be said that the graph theoretical metrics are much more stable and robust and do not show significant differences with kernel size. Overall, it can be noted that when the number of connections, strength, and graph theoretical metrics are considered together, the suggested kernel size is the fwhm with 6 or 8 mm.

Here, one of the points worth mentioning could be the sample size. As is known, performing fMRI studies is challenging in many ways, including technical difficulties, data complexity, participant recruitment, ethics and safety, cost, data interpretation, etc. Among these, one of the main limitations is the high cost of an fMRI scan, which can limit the number of participants. In this context, the sample size of the study should be determined optimally. It should be statistically significant and large enough to generalize to a population but not so large as to be prohibitively expensive or time-consuming. Therefore, the power analysis method is used for the determination of the sample size. In this study, the power of the population was measured over 80%. Since the results of the analyses reflect the group averages and the power of the population is significant enough, it can be foreseen that the results of the wider sample size will approximate the proposed results. In general, it has been reported that the average sample size of most neuroimaging studies is below 25. Moreover, although the average increases to ~0.74 participants per year, it is still under 15 subjects in the most cited studies [46]. Table 2 provides a comparative summary of related studies. The challenges in recruiting fMRI data and data loss due to several reasons, such as high motion, generally results in relatively few valid sample size, which is under 25. Here, it is important to note that the appropriate sample size may vary depending on the research question, the type of study, and other factors.

In summary, the selection of smoothing kernel size may still depend on the hypothesis of the research, spatial resolution requirements, and anatomical features of the data being analyzed. However, the results show that smoothing can yield more consistent analysis results, especially in functional network connectivity. Thus, it should also be kept in mind that smoothing is data-dependent and may cause significant alterations in group-level analysis when it is not applied appropriately.

In future work, the region-specific kernel size estimation problem can be considered. With machine learning algorithms trained according to the selected or marked regions in the brain, the suggestion of the most appropriate kernel size would be ensured. This future study, which requires rigorous and tailored analyses, would also be promising in practical clinical use.

## Figures and Tables

**Table 1:** Graph measure results for functional connectivity analysis of rs-fMRI and tb-fMRI with different kernel sizes.

Kernel (*FWHM*)	Resting					Encoding				
	$E_{glob}$	$E_{loc}$	BC	CC	$l_{avg}$	$E_{glob}$	$E_{loc}$	BC	CC	$l_{avg}$
0 mm	0.65288	0.78633	0.02614	0.60200	1.78437	0.80481	0.87310	0.01305	0.75113	1.39245
2 mm	0.65357	0.78252	0.02639	0.60905	1.79186	0.80388	0.87275	0.01327	0.75002	1.40063
4 mm	0.65361	0.78253	0.02640	0.60915	1.79193	0.79424	0.86558	0.01378	0.73639	1.41412
6 mm	0.65361	0.78253	0.02639	0.60916	1.79192	0.80447	0.86554	0.01378	0.73650	1.41443
8 mm	0.65393	0.77929	0.02623	0.60959	1.78689	0.80488	0.86625	0.01295	0.74221	1.40784
10 mm	0.65589	0.78638	0.02614	0.61200	1.78437	0.80492	0.87316	0.01286	0.74183	1.40638
Average	0.65392	0.78326	0.02629	0.60884	1.78855	0.80287	0.86939	0.01328	0.74301	1.40598

**Table 2:** Sample rs-fMRI and tb-fMRI studies with sample sizes under 25.

Publication	#Subjects	Specification	Description
Somatotopy of cervical dystonia in motor-cerebellar networks: Evidence from resting state fMRI [47]	18	Resting	11 min resting state, in which the gaze monitored with eye tracking
Gender differences in brain regional homogeneity of healthy subjects after normal sleep and after sleep deprivation: A resting-state fMRI study [48]	16	Resting	Resting state for 30 min
Single-subject manual independent component analysis and resting state fMRI connectivity outcomes in patients with juvenile absence epilepsy [49]	8	Resting	Resting state about 14 min
Improving reliability of subject-level resting-state fMRI parcellation with shrinkage estimators [50]	20		Resting state for 7 min
Hemodynamic timing in resting-state and breathing-task BOLD fMRI [51]	9	Resting	Resting state for 7 min 48 s
	9	Task-based +Resting	Breath-hold task and resting-state consecutively
Estimating sample size in functional MRI (fMRI) neuroimagingstudies: Statistical power analyses [52]	6	Resting	Resting state with open eyes for 4 min.
	12	Task-based	A verbal working memory task
Different memory patterns of digits: a functional MRI study [53]	22	Task-based	Short-term, Long Term and Working Memory on numerical figures
A dataset of human fMRI/MEG experiments with eye tracking for spatial memory research using virtual reality [54]	12	Task-based	Eye tracking task for spatial memory
Incidental encoding task [55]	18	Task-based	Participants create new memories without purposely by working in their environment and picking up information in the process.

## Data Availability

The dataset will be available upon request on reasonable request.

## Supplementary Materials

The following supporting information can be downloaded at: https://www.mdpi.com/article/10.3390/s23135866/s1, Figure S1: The Detailed Graphics of Figure 9. Functional connectivity networks as connectome ring for all fwhm kernel sizes.

## Appendix A

The major brain networks and their high cognitive areas that are used as ROIs in the paper are given in detail as follows:

**Default Mode Network (DMN)**	
Medial Prefrontal Cortex	DMN.MPFC
Lateral Parietal left	DMN.LP l
Lateral Parietal right	DMN.LP r
Posterior Cingulate Cortex	DMN.PCC
**Visual Network (VN)**	
Medial Visual area	VN.Medial
Occipital Visual area	VN.Occipital
Lateral Visual area left	VN.Lateral l
Lateral Visual area right	VN.Lateral r
**Dorsal Attention Network (DAN)**	
Frontal Eye Fields left	DAN.FEF l
Frontal Eye Fields right	DAN.FEF r
Intraparietal Sulcus left	DAN.IPS l
Intraparietal Sulcus right	DAN.IPS r
**Sensori Motor Network (SMN)**	
Lateral Sensorimotor area left	SMN.Lateral l
Lateral Sensorimotor area right	SMN.Lateral r
Superior Sensorimotor area	SMN.Superior
**Language Network (LN)**	
Inferior Frontal Gyrus left	L.IFG l
Inferior Frontal Gyrus right	L.IFG r
Posterior Superior Temporal Gyrus left	L.pSTG l
Posterior Superior Temporal Gyrus right	L.pSTG r
**Fronto Parietal Network (FPN)**	
Lateral Prefrontal Cortex left	FPN.LPFC l
Posterior Parietal Cortex left	FPN.PPC l
Lateral Prefrontal Cortex right	FPN.LPFC r
Posterior Parietal Cortex right	FPN.PPC r
**Salience Network (SN)**	
Anterior Cingulate Cortex	SN.ACC
Anterior Insula left	SN.AInsula l
Anterior Insula right	SN.AInsula r
Rostral Prefrontal Cortex left	SN.RPFC l
Rostral Prefrontal Cortex right	SN.RPFC r
SupraMarginal Gyrus left	SN.SMG l
SupraMarginal Gyrus right	SN.SMG r
**Cerebellar Network (CN)**	
Cerebellum Anterior	C.Anterior
Cerebellum Posterior	C.Posterior

## References

[1] Carter R. (2019). The Human Brain Book: An Illustrated Guide to Its Structure, Function, and Disorders.

[2] Bandettini P.A., Birn R.M., Donahue K.M. (2000). Functional MRI: Background, methodology, limits, and implementation. Handbook of Psychophysiology.

[3] Logothetis N.K. (2008). What we can do and what we cannot do with fMRI. Nature.

[4] Cascino G. (2002). Functional MRI for Language Localization. Epilepsy Curr..

[5] Zhang S., Li X., Lv J., Jiang X., Guo L., Liu T. (2016). Characterizing and Differentiating Task-based and Resting State FMRI Signals via Two-stage Sparse Representations. Brain Imaging Behav..

[6] Goebel R., Stippich C. (2007). Localization of Brain Activity using Functional Magnetic Resonance Imaging. Clinical Functional MRI: Presurgical Functional Neuroimaging.

[7] Bijsterbosch J., Smith S.M., Beckmann C.F. (2017). An Introduction to Resting State fMRI Functional Connectivity.

[8] Sporns O. (2010). Networks of the Brain.

[9] Power J.D., Cohen A.L., Nelson S.M., Wig G.S., Barnes K.A., Church J.A., Vogel A.C., Laumann T.O., Miezin F.M., Schlaggar B.L. (2011). Functional network organization of the human brain. Neuron.

[10] Farahani F.V., Karwowski W., Lighthall N.R. (2019). Application of Graph Theory for Identifying Connectivity Patterns in Human Brain Networks: A Systematic Review. Front. Neurosci..

[11] Wang J., Zuo X., He Y. (2010). Graph-based network analysis of resting-state functional MRI. Front. Syst. Neurosci..

[12] Lv H., Wang Z., Tong E., Williams L.M., Zaharchuk G., Zeineh M., Goldstein-Piekarski A.N., Ball T.M., Liao C., Wintermark M. (2018). Resting-State Functional MRI: Everything That Nonexperts Have Always Wanted to Know. Am. J. Neuroradiol..

[13] Friston K.J. (2011). Functional and Effective Connectivity: A Review. Brain Connect..

[14] Cribben I., Wager T.D., Lindquist M.A. (2013). Detecting functional connectivity change points for single-subject fMRI data. Front. Comput. Neurosci..

[15] Calhoun V.D., Adali T. (2016). Time-Varying Brain Connectivity in fMRI Data: Whole-brain data-driven approaches for capturing and characterizing dynamic states. IEEE Signal Process. Mag..

[16] Liu T.T. (2016). Noise contributions to the fMRI signal: An overview. NeuroImage.

[17] Candemir C., Gonul A.S., Selver A.M. (2021). Automatic Detection of Emotional Changes Induced by Social Support Loss using fMRI. IEEE Trans. Affect. Comput..

[18] Aurich N.K., Alves Filho J.O., Marques da Silva A.M., Franco A.R. (2015). Evaluating the reliability of different preprocessing steps to estimate graph theoretical measures in resting state fMRI data. Front. Neurosci..

[19] Vergara V.M., Mayer A.R., Damaraju E., Hutchison K., Calhoun V.D. (2017). The effect of preprocessing pipelines in subject classification and detection of abnormal resting state functional network connectivity using group ICA. NeuroImage.

[20] Strother S.C. (2006). Evaluating fMRI preprocessing pipelines. IEEE Eng. Med. Biol. Mag..

[21] Power J.D., Barnes K.A., Snyder A.Z., Schlaggar B.L., Petersen S.E. (2012). Spurious but systematic correlations in functional connectivity MRI networks arise from subject motion. NeuroImage.

[22] Power J.D., Mitra A., Laumann T.O., Snyder A.Z., Schlaggar B.L., Petersen S.E. (2014). Methods to detect, characterize, and remove motion artifact in resting state fMRI. NeuroImage.

[23] Wu C.W., Chen C.-L., Liu P.-Y., Chao Y.-P., Biswal B.B., Lin C.-P. (2011). Empirical Evaluations of Slice-Timing, Smoothing, and Normalization Effects in Seed-Based, Resting-State Functional Magnetic Resonance Imaging Analyses. Brain Connect..

[24] Shirer W.R., Jiang H., Price C.M., Ng B., Greicius M.D. (2015). Optimization of rs-fMRI Pre-processing for Enhanced Signal-Noise Separation, Test-Retest Reliability, and Group Discrimination. NeuroImage.

[25] Worsley K.J., Friston K.J. (1995). Analysis of fMRI Time-Series Revisited—Again. NeuroImage.

[26] Mikl M., Mareček R., Hluštík P., Pavlicová M., Drastich A., Chlebus P., Brázdil M., Krupa P. (2008). Effects of spatial smoothing on fMRI group inferences. Magn. Reson. Imaging.

[27] Sacchet M.D., Knutson B. (2013). Spatial smoothing systematically biases the localization of reward-related brain activity. NeuroImage.

[28] Murphy J.E., Yanes J.A., Kirby L.A.J., Reid M.A., Robinson J.L. (2020). Left, right, or bilateral amygdala activation? How effects of smoothing and motion correction on ultra-high field, high-resolution functional magnetic resonance imaging (fMRI) data alter inferences. Neurosci. Res..

[29] Molloy E.K., Meyerand M.E., Birn R.M. (2014). The influence of spatial resolution and smoothing on the detectability of resting-state and task fMRI. NeuroImage.

[30] Scheinost D., Papademetris X., Constable R.T. (2014). The impact of image smoothness on intrinsic functional connectivity and head motion confounds. NeuroImage.

[31] Zuo X.-N., Xing X.-X. (2014). Test-retest reliabilities of resting-state FMRI measurements in human brain functional connectomics: A systems neuroscience perspective. Neurosci. Biobehav. Rev..

[32] Wang J., Wang Z., Aguirre G.K., Detre J.A. (2005). To smooth or not to smooth? ROC analysis of perfusion fMRI data. Magn. Reson. Imaging.

[33] Chen Z., Calhoun V. (2018). Effect of Spatial Smoothing on Task fMRI ICA and Functional Connectivity. Front. Neurosci..

[34] Pajula J., Tohka J. (2014). Effects of spatial smoothing on inter-subject correlation based analysis of FMRI. Magn. Reson. Imaging.

[35] Triana A.M., Glerean E., Saramäki J., Korhonen O. (2020). Effects of spatial smoothing on group-level differences in functional brain networks. Netw. Neurosci..

[36] Candemir C. (2023). A Practical Estimation of the Required Sample Size in fMRI Studies. Mugla J. Sci. Technol..

[37] Ebner N.C., Riediger M., Lindenberger U. (2010). FACES—A database of facial expressions in young, middle-aged, and older women and men: Development and validation. Behav. Res. Methods.

[38] Whitfield-Gabrieli S., Nieto-Castanon A. (2012). Conn: A functional connectivity toolbox for correlated and anticorrelated brain networks. Brain Connect..

[39] Poldrack R.A., Mumford J.A., Nichols T.E. (2011). Handbook of Functional MRI Data Analysis.

[40] Silver N.C., Dunlap W.P. (1987). Averaging Correlation Coefficients: Should Fisher’s z Transformation Be Used?. J. Appl. Psychol..

[41] Smith S.M., Hyvärinen A., Varoquaux G., Miller K.L., Beckmann C.F. (2014). Group-PCA for very large fMRI datasets. NeuroImage.

[42] Huettel S.A., Song A.W., McCarthy G. (2014). Functional Magnetic Resonance Imaging.

[43] Ma X., Jiang G., Fu S., Fang J., Wu Y., Liu M., Xu G., Wang T. (2018). Enhanced Network Efficiency of Functional Brain Networks in Primary Insomnia Patients. Front. Psychiatry.

[44] Massullo C., Imperatori C., De Vico Fallani F., Ardito R.B., Adenzato M., Palmiero L., Carbone G.A., Farina B. (2022). Decreased brain network global efficiency after attachment memories retrieval in individuals with unresolved/disorganized attachment-related state of mind. Sci. Rep..

[45] Watts D.J., Strogatz S.H. (1998). Collective dynamics of ‘small-world’ networks. Nature.

[46] Szucs D., Ioannidis J.P.A. (2020). Sample size evolution in neuroimaging research: An evaluation of highly-cited studies (1990–2012) and of latest practices (2017–2018) in high-impact journals. NeuroImage.

[47] Zito G.A., Tarrano C., Jegatheesan P., Ekmen A., Béranger B., Rebsamen M., Hubsch C., Sangla S., Bonnet C., Delorme C. (2022). Somatotopy of cervical dystonia in motor-cerebellar networks: Evidence from resting state fMRI. Parkinsonism Relat. Disord..

[48] Dai X.-J., Gong H.-H., Wang Y.-X., Zhou F.-Q., Min Y.-J., Zhao F., Wang S.-Y., Liu B.-X., Xiao X.-Z. (2012). Gender differences in brain regional homogeneity of healthy subjects after normal sleep and after sleep deprivation: A resting-state fMRI study. Sleep Med..

[49] Parsons N., Bowden S.C., Vogrin S., D’Souza W.J. (2020). Single-subject manual independent component analysis and resting state fMRI connectivity outcomes in patients with juvenile absence epilepsy. Magn. Reson. Imaging.

[50] Mejia A.F., Nebel M.B., Shou H., Crainiceanu C.M., Pekar J.J., Mostofsky S., Caffo B., Lindquist M.A. (2015). Improving reliability of subject-level resting-state fMRI parcellation with shrinkage estimators. NeuroImage.

[51] Gong J., Stickland R.C., Bright M.G. (2023). Hemodynamic timing in resting-state and breathing-task BOLD fMRI. NeuroImage.

[52] Desmond J.E., Glover G.H. (2002). Estimating sample size in functional MRI (fMRI) neuroimaging studies: Statistical power analyses. J. Neurosci. Methods.

[53] Nie J., Zhang Z., Wang B., Li H., Xu J., Wu S., Zhu C., Yang X., Liu B., Wu Y. (2019). Different memory patterns of digits: A functional MRI study. J. Biomed. Sci..

[54] Zhang B., Naya Y. (2022). A dataset of human fMRI/MEG experiments with eye tracking for spatial memory research using virtual reality. Data Brief.

[55] Incidental Encoding Task (Posner Cueing Paradigm). https://openfmri.org/dataset/ds000110/.

