# Supplementary material for: Spatial Smoothing Effect on Group-Level Functional Connectivity during Resting and Task-Based fMRI
Source: Sensors (Basel). 2023 Jun 24;23(13):5866. doi: 10.3390/s23135866 (PMC10346894; doi:10.3390/s23135866)
Supplement: Supplementary file 1 [file sensors-23-05866-s001.zip › sensors-2418569-supplementary.pdf]

Supplementary File

Original Article Title: ***"Spatial Smoothing Effect on Group Level Functional Connectivity During Resting and Task-based fMRI"***

The Detailed Graphics of Figure S1. Functional connectivity networks as connectome ring for all fwhm kernel sizes

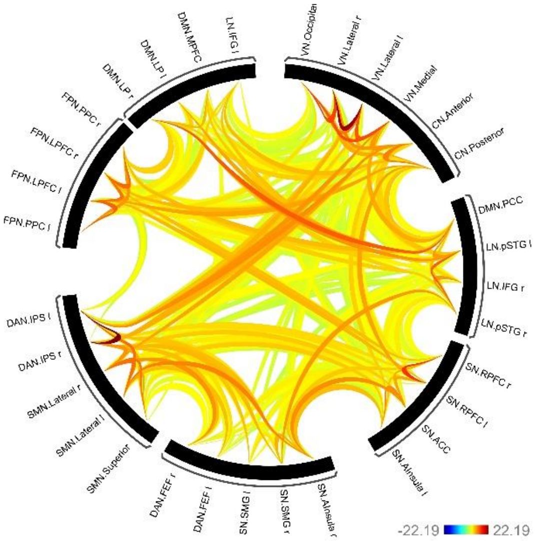

tb-fMRI 0 mm

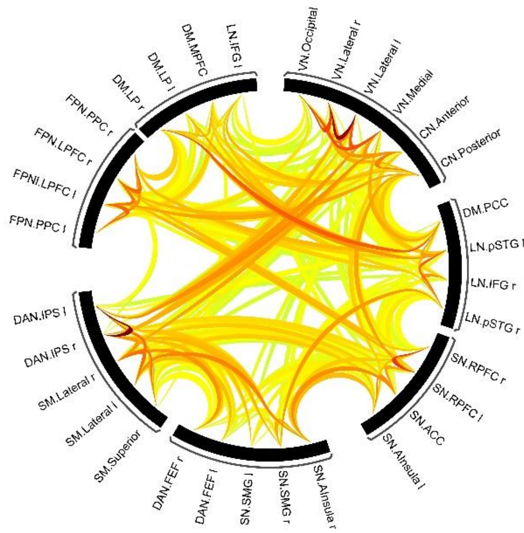

tb-fMRI 2 mm

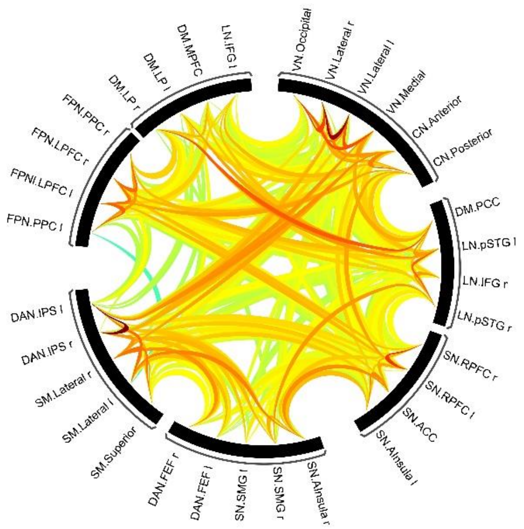

tb-fMRI 4 mm

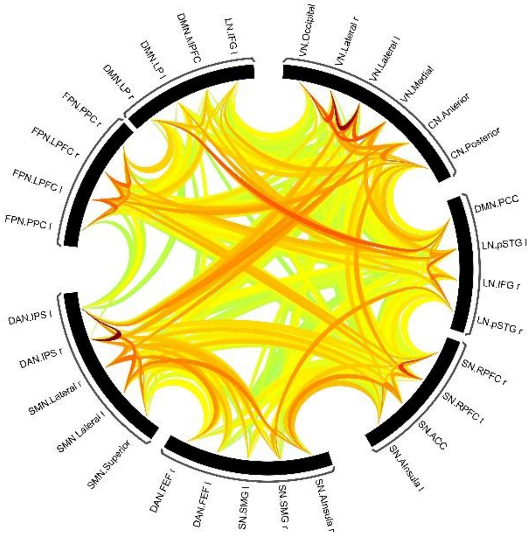

tb-fMRI 6mm

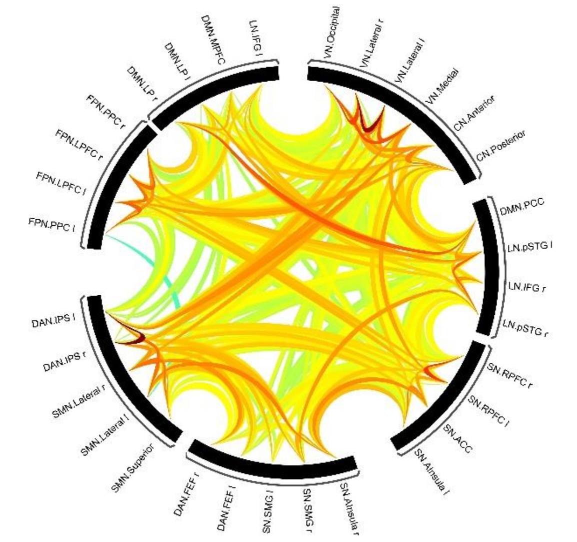

tb-fMRI 8 mm

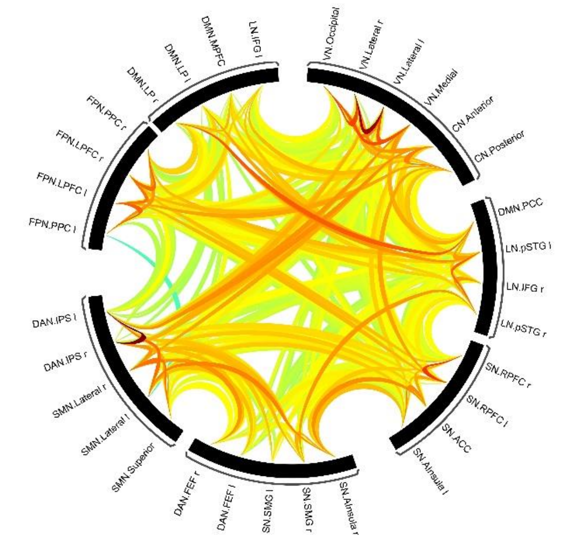

tb-fMRI 10 mm

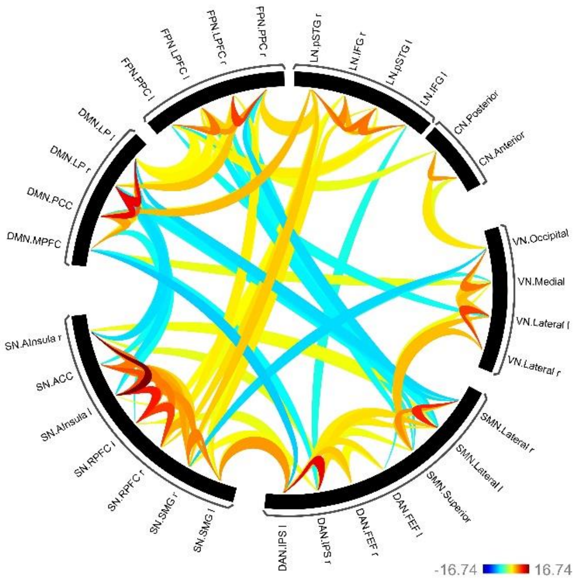

rs-fMRI 0 mm

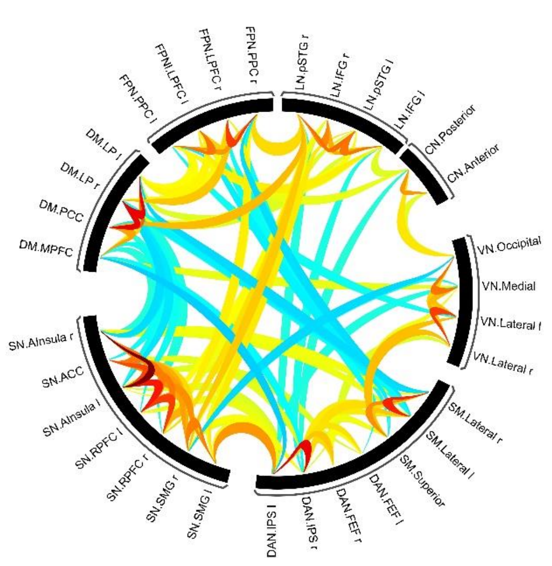

rs-fMRI 2 mm

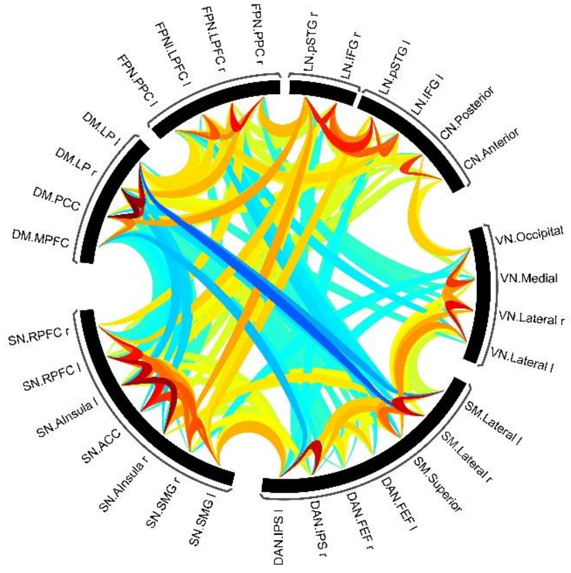

rs-fMRI 4 mm

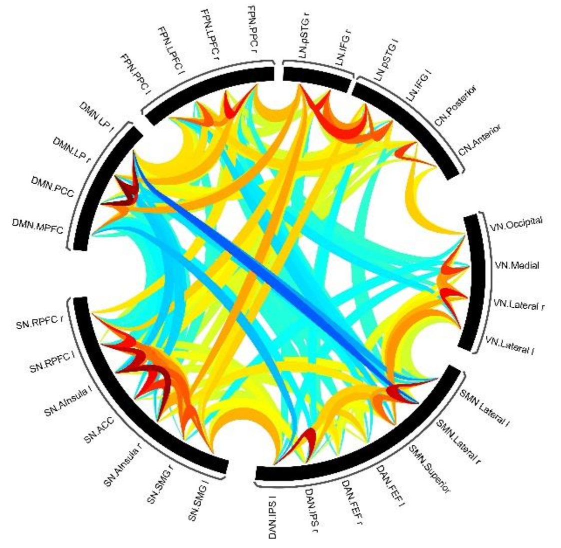

rs-fMRI 6mm

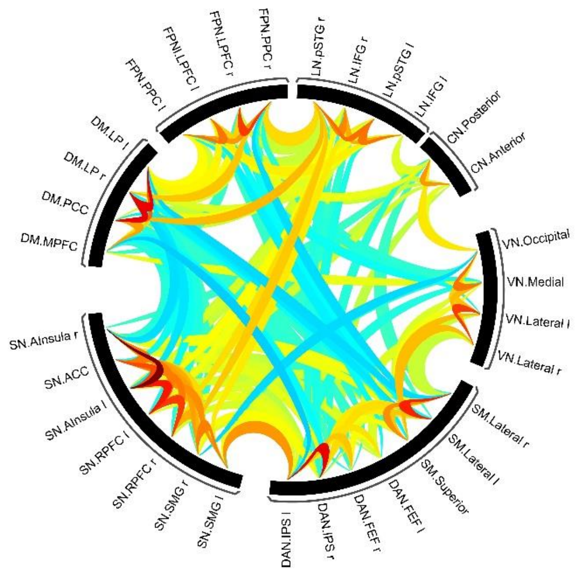

rs-fMRI 8 mm

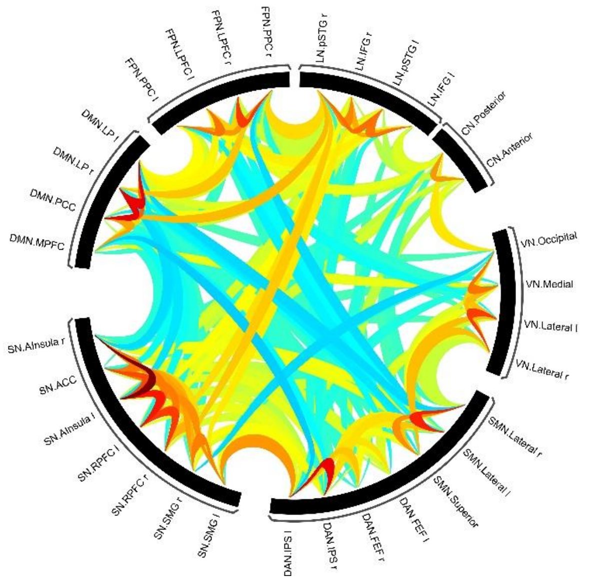

rs-fMRI 10 mm
